# Mortality and cause-of-death reporting and analysis systems in seven pacific island countries

**DOI:** 10.1186/1471-2458-12-436

**Published:** 2012-06-13

**Authors:** Karen L Carter, Chalapati Rao, Alan D Lopez, Richard Taylor

**Affiliations:** 1 School of Population Health, University of Queensland, Herston, Australia; 2 Statistics for Development Programme, Secretariat of the Pacific Community, Noumea, New Caledonia; 3 School of Public Health and Community Medicine, University of New South Wales, New South Wales, Australia

**Keywords:** Mortality, Death registration, Vital statistics, Cause of death, Pacific islands.

## Abstract

**Background:**

Mortality statistics are essential for population health assessment. Despite limitations in data availability, Pacific Island Countries are considered to be in epidemiological transition, with non-communicable diseases increasingly contributing to premature adult mortality. To address rapidly changing health profiles, countries would require mortality statistics from routine death registration given their relatively small population sizes.

**Methods:**

This paper uses a standard analytical framework to examine death registration systems in Fiji, Kiribati, Nauru, Palau, Solomon Islands, Tonga and Vanuatu.

**Results:**

In all countries, legislation on death registration exists but does not necessarily reflect current practices. Health departments carry the bulk of responsibility for civil registration functions. Medical cause-of-death certificates are completed for at least hospital deaths in all countries. Overall, significantly more information is available than perceived or used. Use is primarily limited by poor understanding, lack of coordination, limited analytical skills, and insufficient technical resources.

**Conclusion:**

Across the region, both registration and statistics systems need strengthening to improve the availability, completeness, and quality of data. Close interaction between health staff and local communities provides a good foundation for further improvements in death reporting. System strengthening activities must include a focus on clear assignment of responsibility, provision of appropriate authority to perform assigned tasks, and fostering ownership of processes and data to ensure sustained improvements. These human elements need to be embedded in a culture of data sharing and use. Lessons from this multi-country exercise would be applicable in other regions afflicted with similar issues of availability and quality of vital statistics.

## Background

Accurate mortality statistics are essential for population health assessment, and to design and monitor health intervention programs. Despite extensive collection of health data in Pacific island countries, these data are rarely analysed or utilised [1], as they are considered to be incomplete or unreliable [2-4].

Despite the data shortcomings, there is a general appreciation that non-communicable diseases are increasingly contributing to premature adult mortality in Pacific Island Countries [2,5]. Cancer and cardiovascular disease have been recognised as important causes of death in these countries since the 1980s [6-10], and obesity rates are amongst the highest in the world [10]. Timely and reliable data on mortality and causes of death is needed to effectively monitor and combat this impending epidemic. The ideal mortality data source is an efficient death registration system with medical certification of cause-of-death. In Pacific Island Countries, small population sizes make complete routine registration an attractive proposition, but there is a concomitant challenge of servicing widely dispersed, low density populations.

Since the late 1990’s there has been an increasing international emphasis on improving health information systems and mortality reporting [11-13]. More recently, the Health Metrics Network has highlighted the paucity of vital statistics in developing countries [14]. The Millennium Development Goals (MDG’s) have also pressured governments to demonstrate progress towards these targets [15]. Despite this increased attention, little information is available on the registration and statistical systems in Pacific Island Countries, and the influence of these processes on data quality.

This paper examines the structure and operations of routine death reporting systems in seven Pacific Island Countries; Fiji, Kiribati, Nauru, Palau, Solomon Islands, Tonga and Vanuatu. Strengths and limitations common across national systems are identified, and system characteristics are related to data availability and quality. This review also makes specific recommendations to strengthen these systems and improve data availability and quality at the national and regional level.

## Methods

Countries were selected in order to represent Pacific Island States with a range of: population and land sizes, government structures, distribution of health services, and economic development [1] [see Additional file 1. These countries represent all three regions of the Pacific Islands: Melanesia (Fiji, Solomon Islands, Vanuatu, and Kiribati), Micronesia (Palau and Nauru) and Polynesia (Tonga). A standard analytical framework [16] was used to assess practices for data collection, management, analysis, reporting and utilisation in each country.

Assessments were conducted through collaboration between the authors and key national stakeholders, including representatives from the Ministry of Health and Bureau of Statistics. Other stakeholders represented included: government departments such as the civil registry office, finance, and central planning agencies; local non-government organisations; and country offices of WHO, UNICEF and UNFPA.

The assessment comprised a field visit guided by a detailed data collection tool [16], and involved comprehensive document review, observation of procedures and processes, discussions with staff across organisations and levels, and examination of available mortality data. Informal interview respondents were asked to discuss their role in data collection, management or use, and perceptions of the strengths and weaknesses of the system. Where practices differed from policy or management perspectives, both were documented. For each country, a flow chart was assembled to depict the reporting process from the initial recording of each death to its representation in a national statistical dataset.

All information was subsequently reviewed using a standard assessment framework that classifies performance and design aspects [16] of the system according to the socio-political environment, national administrative framework, system administration, technical characteristics and ownership [16]. The first two refer to the overall context in which routine death reporting is carried out within the country, while the final three are system specific. This framework was adapted from earlier approaches [17-19] with the additional assessment of three human elements that are central to system performance in the Pacific Islands; responsibility and authority under administrative aspects of the system, and organisational ownership [16].

Country and system specific findings were distilled into key themes that reflect strengths and weaknesses common to multiple systems across the countries reviewed, that have the potential to significantly impact the coverage, completeness and quality of data, or its access and utility. Finally, potential impacts of these system characteristics were reviewed in relation to available data.

## Results and discussion

### Common elements of routine reporting systems

**Figure 1:**
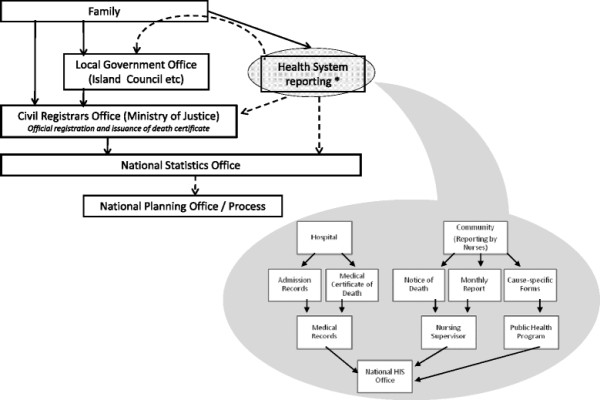
**Routine Death Reporting Pathways in the Pacific Islands. ***Dotted lines indicate link does not apply to all study countries.*

Routine reporting of deaths in the Pacific Island countries is predominantly managed by civil registration systems or Health departments (Figure 1). The importance of health reporting systems in supporting civil registration was evident across countries. In particular, despite existing legislation, civil registration of deaths was not active in the Solomon Islands or Vanuatu.

Within health departments, deaths are reported by medical practitioners and community nursing staff (Figure 1). Medical certification of death is practiced in hospitals in all countries, however data from death certificates is routinely collated only in Fiji, Nauru, Palau, Tonga, and in Port Vila hospital in Vanuatu. Collated data is coded according to the International Classification of Disease (ICD) version 10 in Fiji, Tonga and Port Vila hospital, while Nauru has subsequently started coding since this time. Until recently only hospital discharge data was coded in Palau (using ICD version 9), although recent changes subsequent to this review have seen the introduction of coding for medical certificates of death using ICD 9. In Kiribati, the Solomon Islands, and other hospitals in Vanuatu, data for hospital deaths is compiled from discharge data and coded according to ICD version 10.

In Fiji, Palau, and Nauru, medical certification is also conducted for deaths occurring in the community. In all countries except Nauru, community deaths are reported through monthly nursing reports. These are only routinely collated in Fiji, Kiribati and Vanuatu. Reporting through the hospitals accounts for nearly all reported deaths in the small islands of Palau and Nauru, and a high proportion of reported deaths in Tonga; while a much larger proportion are reported through monthly nursing reports in the other countries. Only in Kiribati is there a large proportion of deaths reported through civil registration that are not also reported *via* the health system.

Other routine death recording systems in these countries are operated by churches, cemeteries and the police. However, these additional sources have limited application in improving the completeness of death records as they pertain only to specific sub-groups within the population, the records are of doubtful quality, or the data cannot be easily extracted.

### Key strengths and weaknesses

Strengths and weakness of the routine reporting systems are reviewed below, with key themes common across countries reviewed outlined in Table 1.

**Table 1:** Key Strengths and Weaknesses of Mortality Reporting Systems in the Pacific Islands

**Category**	**Strengths**	**Weaknesses**
Societal Issues	Social incentives for registration	Private land burials without official approval
Administrative Environment	Existing legal framework	Inadequate/inconsistent implementation of laws
	Health systems involvement in Civil registration and vital statistics operations	Passive registration *i.e.* onus to report on citizen
		Registration process for “off-island” deaths unclear
	National statistics committees	Complex statistical reporting requirements
System Issues – Administration	Community nurses formally tasked to notify vital events	Improper emphasis on community nurses to report cause of death
	Routine compilation of mortality data by different health departments	Need for better coordination to generate one reconciled mortality dataset from the health system
		Private health intuitions rarely integrated adequately into reporting systems
		No clear delineation of responsibility across institutions, leading to task duplication
		Personnel lack authority to query/clarify data
System Issues – Technical	Standard international medical death certificate (except Nauru)	Lay reporting of cause for deaths outside facilities in some countries
		Medical certificates of death not routinely tabulated in all countries
	Trained ICD coders	High turnover of trained staff
	Key personnel adequately skilled for data management at national level	Statistical analysis limited to ten leading causes of death
		Insufficient data quality assessment and control
	Initiatives to set data standards for health information	Dysfunctional/outdated software programs not amenable to modification or upgrade
System Issues – Ownership	Strong ownership of systems/interest at national levels that has contributed substantially to ongoing survival of the systems	Generally poor feedback to local level staff
		Many systems are highly dependent on one or two key individuals with a strong interest in providing health data

Source data: [16].

### Societal issues

Initial reporting and formal registration of deaths relies primarily on family members. Social factors such as burial arrangements, public support, and political interest in data for planning purposes therefore have a significant bearing on the likelihood of the death being reported, and the quality of the data captured.

Burial in cemeteries (parts of Vanuatu) require specific approvals, which promotes registration. Such approvals are not necessarily required for burial on private lands (as in Kiribati). Funeral customs that require extended family to gather, as observed in Palau and Tonga also encourage reporting. In these cases, the death certificate facilitates airline reservations, and the body is held for several days at a mortuary. Social incentives for reporting are limited, although access to pension plans or land transfers may encourage reporting, particularly for adult male deaths. A notable exception is Nauru where a government funeral assistance payment strongly encourages universal reporting. Local health personnel are closely integrated into the community across these countries, and are an important foundation for developing public support. Across the region, there is increasing evidence of data use in deciding national health priorities [20-22], with the Pacific Health Ministers meeting in 2009 recommending that countries “improve the quality and reliability of data” [23].

### National administrative environment

The collection and compilation of national mortality and cause-of-death statistics involves multiple government agencies (Figure 1). There is significant potential for duplication of effort and inconsistencies in the data, in the absence of appropriate coordination at the central level. Equally, inadequate delineation of organisational responsibilities could result in significant data gaps.

Legislation requiring registration of deaths exists for all islands [24-31], although not fully implemented, such as in rural areas of Vanuatu [24]). Current practices are also not necessarily reflected in the legislation, as in Fiji where deaths cannot be registered prior to burial or cremation. While all countries have a process for holding an inquest or inquiry for a death suspected to have resulted from a criminal act, fire or other causes of specific interest, only Fiji and Kiribati have legislation that defines responsibility for ensuring findings are subsequently forwarding to the civil registry and used to complete the registration process [25,26]. In Vanuatu and the Solomon Islands, health systems have evolved to perform the role of civil registration. Compilation of cause-of-death data was generally recognised as a role of the health department despite not being defined in legislation, except in Fiji where coding was duplicated at the Bureau of Statistics. All countries currently have functional inter-agency committees to meet reporting requirements for the Millennium Development goals [15]. These have had a clear impact in improving coordination and collaboration between departments.

### System issues – administration

Compilation of mortality statistics also involves a wide array of organisational units within and across agencies (Figure 1). In the larger countries, provincial government structures add to this complexity (Additional file 1). The operations and subsequent data quality are therefore influenced by administrative aspects within the system.

In most countries, the system design was appropriate for available resources, with a clear mandate for data compilation. However, in Vanuatu, an over-reliance on technology was noted, that did not have the requisite infrastructure. In most departments, responsibilities for reporting and analysis of data were not clearly assigned; or, were assigned to staff without adequate training, resulting in poor use of existing data sets. A strong demand for health information in Palau, Tonga and Kiribati translates to better access for data managers to query or clarify issues with doctors and senior staff regarding the data. In contrast, data managers in Vanuatu, the Solomon Islands, and Fiji had limited authority, either real or perceived, to raise concerns. In decentralised civil registration systems, local registrars are not directly under the authority of the national Civil Registrar, leading to confusion in the processes for resolving issues.

### System issues – technical

Duplication of data compilation was observed in all countries. This was particularly apparent between provincial and national systems (for both health and civil registration); and in the parallel reporting systems for specific health programs (Figure 1). For example, community nurses in the Solomon Islands use up to nine forms to report a death. Databases that cannot be modified, or which use obsolete software for which technical support is no longer available, as seen in Vanuatu and Kiribati, contribute to the proliferation of multiple databases to meet changing needs. The international standard medical certificate of death [32] is used in all countries except Nauru. Much of the collected data was not available for analysis, such as in Kiribati where the health department does not retain a complete copy of the certificate. Quality control processes are limited, except in Palau where completed medical certificates are routinely reviewed by senior doctors resulting in a low proportion of deaths being assigned to ill-defined causes. Recording of infant deaths was inconsistent as result of unclear procedures, a lack of data standards, and complicated forms; with early deaths potentially missed (Kiribati) or stillbirths included in the data (Nauru).

In Fiji and Tonga, data entry and coding practices leading to alterations in the causal sequence on the medical certificate result in difficulties in applying ICD rules to select the underlying cause [32]. A high staff turnover for doctors (Nauru, Solomon Islands and Kiribati), nurses (Fiji) and key analytical roles (Tonga and Fiji) has also adversely affected knowledge of reporting processes and depleted the skill set available in some areas.

### System issues – ownership

A sense of ownership in individuals as well as institutions is essential for smooth operation of vital registration systems. In general, a broader sense of shared ownership provides the system with greater flexibility to respond to changed circumstances such as staff turnover or natural disasters.

Feedback to staff is an important mechanism to foster ownership among personnel at different levels. This has been provided through annual public health conferences, (*e.g.* Palau, Tonga and the Solomon Islands); and field visits by data managers (*e.g.* Tonga and the Solomon Islands). In Tonga, such visits include local civil registrar staff and community leaders. At local levels, there is considerable variation in the quality of registers and submission of statistical returns by community nurses in Kiribati, Solomon Islands and Vanuatu, which reflects the degree of ownership perceived by them. At the same time, a strong sense of ownership at the national level of the health information system in Vanuatu helped sustain the overall data compilation process. In health facilities, physicians tended to store medical records for deaths separately, to preserve them from loss or destruction. This practice limits access to such data for reporting and analysis.

### Data availability

As shown in Table 2, significantly more data was identified as available for reporting and analysis than previously recorded by international sources. Additional data sources (Figure 1) that were not tabulated (and therefore not accessible for analysis) were also found.

**Table 2:** Tabulated mortality data available for selected Pacific Islands, 2000–2009: WHO databases, as compared with locally available data

		**Mortality data by age and sex**		**Cause of death data**	
**Country**	**Estimated Deaths (2011)**^**#**^	WHO* (Year/Completeness)	**Locally available data**^**+**^**(estimated completeness**^$^**)**	**WHO**^**§**^**(Year/Quality)**	**Locally available data**^**+**^**(from medical certificates)**^**+**^
Fiji	7185	No data (1999/90-100%)	MoH reports (>95%)	2000/Low	MOH reports
Kiribati	827	2001/>75%	MoH reports (40-60%) Civil registration (40-60%)	2002/Low	Not tabulated
Nauru	88	No data	Civil Registration (>95%)	1996/not rated	MoH reports
Palau	158	No data	MoH reports (>95%)	No data	MoH reports
Solomon Islands	4039	No data	MoH reports (not estimated)	No data	Not tabulated
Tonga	683	No data	MoH^ reports (>80%)	1998/Low	MoH reports
Vanuatu	1311	No data	MoH reports (not estimated)	No data	MoH reports (hospital deaths only)

^#^ Estimated deaths extracted from Secretariat of the Pacific Islands Population data [33].

* Data extracted from World Health Organisation Statistical Information System [34], [35-40]: data last updated March 2011.

+ Most complete source listed only. MoH = Ministry of Health. Most complete available source or reconciled data: Fiji (MoH), Kiribati (reconciled MoH and civil registry data), Solomon Islands (MoH), Vanuatu (MoH), Nauru (MoH/Civil registry data), Palau (MoH), Tonga (MoH).

^$^ Estimates of completeness source: Fiji [41], Kiribati (capture-recapture analysis, unpublished), Solomon Islands (system assessment), Vanuatu (system assessment), Nauru [42], Palau (Brass analysis), Tonga [43].

^**§**^ WHO assessment, 2003 [3].

^ MoH data from 2009 onward is routinely reconciled against civil registry data.

System characteristics indicate that under-registration of deaths is likely from civil registration data sets excluding Nauru [42] and Palau. Health department death reporting systems were estimated to be more than 90% complete in Fiji [41] and Palau (Table 2), and sufficiently complete in Tonga to produce reliable estimates of mortality when corrected for under-reporting [43]. ICD coded [32] cause-of-death data based on medical death certificates were available for both hospital and community deaths in Fiji, and Tonga, with un-coded data based on medical certificates available for Nauru and Palau. Very few deaths in Palau are assigned to ill-defined causes, due to quality control measures described previously. Data from both civil registration and health reporting systems in Kiribati appear incomplete, although a reconciled data set derived from both systems would be more plausible. Only Vanuatu and the Solomon Islands did not have routine reporting systems capable of generating estimates of the mortality level. All three had systems from which cause-of-death could be extracted for hospital deaths; with some cause-of-death information available for deaths in the community, either based on family or community nurses reports.

## Conclusions

Despite the data gaps identified in this analysis, significantly more information on mortality of populations in the Pacific is available than currently used (Table 2). Findings suggest that while significant investment is required to improve capture of cause-of-death, it should be possible for all the countries reviewed to be able to generate reliable data on mortality level within 4–5 years. Causes of death reported by nurses are currently of limited value due to use of broad categories and high proportions of "ill-defined" causes. The current system is appropriate to record only demographic aspects of death, unless procedures such as verbal autopsy [44] are introduced. This should be considered for systems such as Kiribati, Solomon Islands and Vanuatu where universal medical certification is not currently feasible. All of the health department reporting systems reviewed were affected by problems with data extraction, duplicate data sets, coding and data entry issues and difficulties accessing records in the database that adversely affect data quality.

Legislation requiring registration of deaths exists for all islands, but does not necessarily reflect current practices. Health departments are carrying the bulk of responsibility for supporting routine data collection and civil registration functions, and their importance cannot be overemphasised. Efforts to improve data collection, use, and acceptability to decision makers will need to either focus on these systems, or ensure their integration into official reporting processes. A clear strength in all countries reviewed was the close interaction between health staff and local communities, including the opportunities this creates for building strong reporting relationships at the local level.

Significant duplication of data collection and entry exists across all systems, reflecting issues with data ownership, obsolete and unresponsive technology and fractured management of the reporting systems. The assessment identified three critical human elements that influence the effectiveness of civil registration and vital statistics operations. These are responsibility, authority to effectively undertake assigned responsibilities, and ownership or the value that staff place on the reporting system and their role within it.

The approach used in this study shares many similarities with the comprehensive assessment tool for vital statistics systems developed by researchers at the University of Queensland and WHO [19] which is primarily a policy tool that aims to lead countries through a critical self-appraisal of their systems as a basis for developing a planning document for system improvement. The framework presented here was developed from a similar history, however is intended to provide a structured critical evaluation of system characteristics to inform the interpretation of available and published data from the region, yet also allows findings to be used in a policy setting to guide decisions around future system improvements required. Although conducted in partnership with the Ministry of Health and Bureau of Statistics, and through close collaboration with other local partners; the assessment reported here was led by researchers external to the systems being evaluated. This could potentially result in less ownership of the findings by those in a position to effect system improvements. However this approach allows an assessment by objective observers, with a broad range of experience across different reporting systems, and using a standardised framework; thus subsequently allowing for a regional comparison and for common themes to be distilled from individual country data. Further, this approach allows staff at all levels to discuss their views privately, and provides an opportunity for in-depth review where resources may not otherwise be available.

While many of the systems reviewed were highly complex, the framework used in this study provides a useful means of identifying themes for further consideration. This paper is designed to provide an insight into the mortality and cause-of-death reporting in the region. Several of the issues are identified here for the first time. Although previous studies have been limited, the issues raised are consistent with those noted in this assessment. These include little incentive to register vital events [45] particularly in the case of children [46], a lack of any “attempt to estimate and correct for under-enumeration” [2], and referral biases in cause-of-death analysis due to the reliance on hospital data in the absence of robust community based reporting [47].

Across the region, both registration and statistics systems need strengthening to improve the access, completeness, and quality of data. Close interaction between health staff and local communities provides a good foundation for further improvements in death reporting. Suggested priorities to strengthen systems are noted in Table 3. Addressing issues of authority and support, and reducing the duplication of functions across systems would assist in alleviating resource pressure. Longer term priorities relate to broader shifts in practice and would require significant investment.

**Table 3:** Key Regional Priorities for Action

**Assessment Category**	**Recommendations**
Societal Issues	Recognise the importance of community nurses in the reporting process (including links and influence with the local community) and provide support for increased training and feedback on how their data is used in health planning. [Long term]
Administrative Environment	Conduct operational research to validate reporting completeness and quality in functioning systems to build internal management support and confidence to use results. [Short Term]
	Formalise MOU or similar between health departments and civil registration offices on data sharing and cause of death information. [Short Term]
System Issues – Administration	Identify and minimise duplication between parallel systems (particularly within health departments), through data sharing agreements. [Short Term]
	Assign staff (and adequate resources) to analysis role. [Short Term]
	Investigate ways of better engaging provincial governments (island councils, municipal governments etc.) with civil registration, and support this with feedback at a provincial level. [Long Term]
System Issues – Technical	Support countries to identify staff responsible for each process and provide in-country training to support them to meet minimum skill and knowledge level. [Short Term]
	Encourage tabulation and use of medical certificates as a source of information rather than relying solely on hospital discharge data. [Short Term]
	Design databases to capture medical certificates as written. Create an additional field for selection of underlying cause to avoid changes to recorded sequence. [Short Term]
	Encourage use of standard statistical measures (confidence intervals, and rolling averages) and reporting of system limitations to aid in appropriate data interpretation. [Short Term]
	Assess and build staff capacity, particularly in data analysis and certification practices. [Short Term]
System Issues –Ownership	Build supportive relationships between data managers, senior management and doctors that will allow data managers to develop sufficient authority to question data as needed. [Long Term]

Source data: [16].

System strengthening activities must focus on elements such as clear assignment of responsibility, provision of appropriate authority to perform assigned tasks, and fostering ownership of processes and data to ensure sustained improvements. These human elements need to be embedded in a culture of data sharing and use. Although there are common priorities for system improvement across the region, the diversity of infrastructure, social context and existing system capacity will require action to address priorities to be locally appropriate. Governments and international agencies should also support the use of local data wherever possible. Lessons from this multi-country exercise would be applicable in other regions where vital statistics systems are similarly characterised by duplication, inadequately trained staff, and poor use of good quality data on births and deaths in informing policy decisions.

## Conflicts of interest

The authors declare that they have no conflicting interests.

## Authors’ contributions

KLC: developed and trialed the system assessment framework and data collection tools, conducted the field visits and interviews, collated the findings and extracted key themes, and drafted the analysis. CR: assisted with the identification and clarification of key themes, reviewed the data analysis and interpretation of results; revised the manuscript critically for structure, clarity and important intellectual content. AL: revised the manuscript critically for important intellectual content. RT: reviewed the data analysis and interpretation of results; and revised the manuscript critically for important intellectual content. RT and AL both conceived and coordinated the broader study under which this project was undertaken. All authors read and approved the final manuscript.

## Pre-publication history

The pre-publication history for this paper can be accessed here:

http://www.biomedcentral.com/1471-2458/12/436/prepub

## Supplementary Material

Additional file 1 Appendix 1. Country Characteristics Click here for file
