# Supplementary material for: Mortality and cause-of-death reporting and analysis systems in seven pacific island countries
Source: BMC Public Health. 2012 Jun 13;12:436. doi: 10.1186/1471-2458-12-436 (PMC3416646; doi:10.1186/1471-2458-12-436)
Supplement: Additional file 1 — Appendix 1. Country Characteristics [file 1471-2458-12-436-S1.doc]

**Appendix 1: Country Characteristics**

| **Region** | **Melanesia** | | | | **Micronesia** | | **Polynesia** |
| --- | --- | --- | --- | --- | --- | --- | --- |
| **Country** | **Fiji** | **Kiribati** | **Solomon Islands** | **Vanuatu** | **Nauru** | **Palau** | **Tonga** |
| ***Demographics*** |  |  |  |  |  |  |  |
| Population (2009) [1] | 843,888 | 98,989 | 535,007 | 238,903 | 9,771 | 20,397 | 103,023 |
| % of Population under 15 (2009) [1] | 29% | 36% | 41% | 38% | 36% | 21% | 38% |
| ***Geography*** |  |  |  |  |  |  |  |
| Land size (sq. kms)+ [1,2] | 18,272 | 811 | 28,370 | 12190 | 21 | 444 | 650 |
| Capital | Suva | Tarawa | Honiara | Port Vila | Yaren | Melekeok (Business– Koror) | Nuku’alofa |
| No. of inhabited islands (total islands) [2-4] | 106 (332) | 22 (34) | 347 (922) | 65 (83) | (1) | 8 (200) | 36 (170) |
| ***Development*** |  |  |  |  |  |  |  |
| UNDP Development Index Rank (out of 182) (2007) [5] | 108 | Not ranked | 135 | 126 | Not ranked | Not ranked | 99 |
| GDP per capita (USD) (2010)+ [1] | 3,499 | 1,490 | 1,014 | 2,218 | 2,071 | 8,423 | 2,629 |
| Major Industries | Agriculture, fishing and tourism | Subsistence agriculture and fishing | Agriculture | Agriculture and tourism | Mining (phosphate) | Tourism and sale of fishing licenses | Agriculture and tourism |
| ***Government*** |  |  |  |  |  |  |  |
| Government Type | Military | Democratic | Democratic | Democratic | Democratic | Democratic | Monarchy |
| ***Health System*** |  |  |  |  |  |  |  |
| Central vs Provincial | Centralised | Centralised | Provincial | Provincial | Centralised | Centralised | Centralised |
| Number of Hospitals [6] | 3 area hospitals, 76 health centres, 19 sub-divisional medical centres, 3 divisional hospitals and 3 speciality hospitals | 4 (including small facilities) | 8 public, 3 private | 5 public, 1 private | One | One | 4 (1 tertiary and 3 district hospitals) |

**References**

1. SPC. 2009 Pocket Statistical Summary. Noumea: Secretariat of the Pacific Community http://www.spc.int/sdp/index.php?option=com_docman&task=cat_view&gid=28&Itemid=42 Accessed [2 April 2011].
2. ABC Island descriptions http://www.abc.net.au/ra/pacific/places/country/palau.htm Accessed [2 April 2011].
3. Stanley D. South Pacific Organizer. http://www.southpacific.org/guide/tonga.html Accessed [2 April 2011].
4. Ramsar Sites Information Service. Republic of Kiribati http://ramsar.wetlands.org/Portals/15/Republic_of_Kiribati.pdf Accessed [2 April 2011].
5. UNDP. Human Development Report 2009 http://hdrstats.undp.org/en/indicators/87.html Accessed [2 April 2011].
6. Western Pacific Country Health Profiles (CHIPS) 2010 Revision. World Health Organization. Manila; 2008
